# Do CAS measurements correlate with EOS 3D alignment measurements in primary TKA?

**DOI:** 10.1007/s00167-016-4031-3

**Published:** 2016-02-25

**Authors:** Marrigje F. Meijer, Alexander L. Boerboom, Sjoerd K. Bulstra, Inge H. F. Reininga, Martin Stevens

**Affiliations:** 1 Department of Orthopaedics, University of Groningen, University Medical Center Groningen, PO Box 30.001, 9700 RB Groningen, The Netherlands; 2 Department of Trauma Surgery, University of Groningen, University Medical Center Groningen, Groningen, The Netherlands

**Keywords:** Computer-assisted surgery, CAS, Computer navigation, Total knee arthroplasty, Long-leg radiograph, Coronal alignment, Total knee replacement, TKA, TKR

## Abstract

**Purpose:**

Objective of this study was to compare intraoperative computer-assisted surgery (CAS) alignment measurements during total knee arthroplasty (TKA) with pre- and postoperative coronal alignment measurements using EOS 3D reconstructions.

**Methods:**

In a prospective study, 56 TKAs using imageless CAS were performed and coronal alignment measurements were recorded twice: before bone cuts were made and after implantation of the prosthesis. Pre- and postoperative coronal alignment measurements were performed using EOS 3D reconstructions. Thanks to the EOS radiostereography system, measurement errors due to malpositioning and deformity during acquisition are eliminated. CAS measurements were compared with EOS 3D reconstructions. Varus/valgus angle (VV), mechanical lateral distal femoral angle (mLDFA) and mechanical medial proximal tibial angle (mMPTA) were measured.

**Results:**

Significantly different VV angles were measured pre- and postoperatively with CAS compared to EOS. For preoperative measurements, mLDFA did not differ significantly, but a significantly larger mMPTA in valgus was measured with CAS.

**Conclusion:**

Results of this study indicate that differences in alignment measurements between CAS measurements and pre- and postoperative EOS 3D are due mainly to the difference between weight-bearing and non-weight-bearing position and potential errors in validity and reliability of the CAS system. EOS 3D measurements overestimate VV angle in lower limbs with substantial mechanical axis deviation. For lower limbs with minor mechanical axis deviation as well as for mMPTA measurements, CAS measures more valgus than EOS. Eventually the results of this study are of clinical relevance, since it raises concerns regarding the validity and reliability of CAS systems in TKA.

**Level of evidence:**

IIb.

## Introduction

Malalignment in total knee arthroplasty (TKA) leads to increased wear and a higher risk of aseptic loosening, resulting in revision TKA (rTKA) [2, 14, 20, 31, 35]. Malaligned prostheses are associated with inferior clinical results and longer hospital stay [10, 25, 27]. Computer-assisted surgery (CAS) can be used to alignment intraoperatively. There are several techniques to assess alignment pre- and postoperatively.

Goal during TKA is to achieve a neutral mechanical leg axis and to place the femoral and tibial component in neutral alignment [15, 25, 32]. CAS has been developed to improve knee prosthesis alignment and to reduce the number of outliers; multiple studies have shown significant improvement over conventional techniques [3, 4, 8, 9, 19, 28, 39]. The use of CAS during TKA (CAS-TKA) also gives surgeons the possibility to perform reliable intraoperative lower limb alignment measurements [17, 21, 44, 45].

Lower limb alignment measurements are important for both preoperative planning and postoperative evaluation. There are several methods for coronal alignment measurement. Long-leg standing radiographs (LLR) are mostly used in clinical practice to assess coronal alignment pre- and postoperatively. Advantages of this technique are the availability in most centres, low radiation dose and weight-bearing images. A disadvantage is the divergence in the horizontal and vertical planes, which affects the validity of the measurements. Moreover, varus and valgus deformity, rotation and flexion of the leg during acquisition are known to influence coronal alignment measurements, making measurements less valid [6, 23, 26, 33, 40]. CT scan could also be used to overcome these problems, but that technique involves a higher level of radiation, is more costly, and produces non-weight-bearing images.

Several studies have compared intraoperative imageless CAS measurements with pre- and postoperative LLR measurements [1, 18, 37, 42, 43]. Willcox et al. [42] showed that there are discrepancies between intraoperative CAS measurements and those performed on LLRs. The radiological measurements tended to show a larger deformity than CAS measurements. Babazadeh et al. [1] compared alignment measurements of LLR, CT scan and CAS and found that measurements of LLRs and CT were well correlated but little agreement existed between CAS measurements and the two modalities. Reasons for this could be that the CAS measurements are non-weight-bearing, the capsule is unclosed, and the system itself is subject to observer error [1, 42]. Discrepancies between CAS and LLR measurements can also be based on the variability of alignment measurements due to limb malpositioning during acquisition of LLR. Yaffe et al. [43] found a greater discrepancy between CAS and LLR measurements with larger lower limb deformities. Varus or valgus deformity in combination with malpositioning during acquisition is known to alter coronal alignment measurements on LLRs [40].

The EOS 2D/3D system [13, 22] is a new model-based technique that can be used to perform pre- and postoperative alignment measurements. Advantages of EOS are that it uses 3D software, by which the system mathematically corrects for malpositioning during acquisition; thus, measurements might potentially be more valid [30, 41]. Because the system scans the lower limb using a C-arm, there is no divergence in the vertical plane. Performing coronal alignment measurements both pre- and postoperatively with EOS 3D has been proven to be valid and reliable [16, 29]. With the EOS 3D system, these measurement errors due to malpositioning are eliminated [30, 41]. Also, validity of the images may be improved since divergence in the vertical plane is diminished. A disadvantage of EOS is the fact that it is a new device not widely available yet.

Aim of this study was to compare CAS alignment measurements during the primary TKA procedure with pre- and postoperative coronal alignment measurements using EOS 3D reconstructions. CAS measurements have not been compared with 3D X-ray measurements before. Since CAS measurements are also 3D based, potential differences between the two devices cannot be explained by malpositioning during acquisition. If there are differences, other explanations have to be sought.

## Materials and methods

Data were prospectively collected of patients who underwent primary TKA with CAS (CAS-TKA) using the ORTHOsoft Navitrack system (Zimmer inc., Warsaw, IN, USA) between December 2012 and November 2014. The surgeries were performed by two orthopaedic surgeons who have extensive experience with the use of CAS during TKA.

In this study, 52 primary TKA patients (56 knees) were included. The group consisted of 18 males and 34 females with a mean age of 60 ± 9.6 years (range 36–82): this made 50 knees available to compare CAS measurements to the preoperative as well as the postoperative EOS measurements. Due to errors of the navigation system or because a navigation tracker had to be removed when it blocked surgical instruments, only the first CAS measurement was used in five cases. Also, one patient had a fracture at the location of the tibial tracker; therefore, it was decided to exclude that postoperative EOS measurement. In six cases only the second CAS measurement and postoperative EOS measurement were used. The pre- and postoperative EOS measurements and both CAS measurements were used in 44 cases.

### Procedure

Alignment measurements investigated in this study were:

- Varus/valgus angle of the leg (VV): the angle between the line from the femoral head to the centre of the knee and the line from the centre of the ankle to the centre of the knee in the coronal plane.
- Mechanical lateral distal femoral angle (mLDFA): the angle between the mechanical axis of the femur and the tangent to the distal parts of the condyles in the coronal plane.
- Mechanical medial proximal tibial angle (mMPTA): the angle between the mechanical axis of the tibia and the tangent to the tibial plateau in the coronal plane.

Intraoperative CAS measurements were performed and saved twice: VV, mLDFA and mMPTA were measured before any surgical interventions were performed, and VV was measured again after implantation of the knee prosthesis. VV was measured with the leg in extension and the patella reduced while performing manual axial pressure, mimicking a weight-bearing measurement. The first CAS measurements were compared with the preoperative EOS 3D measurements, and the second CAS measurement was compared with the postoperative EOS 3D measurement.

Anteroposterior (AP) and lateral (LAT) weight-bearing X-rays were taken of all patients pre- and postoperatively using the EOS 2D/3D system (EOS Imaging, Paris, France) as part of the standard TKA protocol. The images were anonymised by removing names and patient numbers. SterEOS software (EOS Imaging, Paris, France) was used to create 3D reconstructions of these AP and LAT images. The 3D reconstructions were performed by one of the authors, who had done >100 EOS 3D reconstructions before the start of this study. Of the preoperative images, 3D reconstructions were performed following the guidelines of the manufacturer. For all angles, a negative (−) value indicated varus and a positive (+) value indicated valgus. Since several landmarks disappear or change when a knee prosthesis is implanted, the adjusted guidelines as described earlier [29] were followed for postoperative 3D measurements. A description of the measurement protocols is added in the “Appendix”. Since the distal femur and proximal tibia were replaced by prosthetic components, only the VV could be measured in 3D on the postoperative images.

In accordance with regulations of the Medical Ethical Review Board of University Medical Center Groningen, patients were informed that data of their CAS measurements and radiographs could be used for scientific research. The data of patients who had objections to the use of their data were not included in the study.

### Statistical analyses

For statistical analysis, IBM SPSS Statistics for Windows software (version 22.0, Armonk, NY: IBM Corp.) was used. Potential differences in means between the CAS and EOS measurements were compared using a paired Student *t* test. Correlations between the CAS and EOS measurements were determined using Spearman’s *ρ* and were interpreted according to the benchmarks described by Domholdt [12]: a *ρ* 0.90–1.00 represents a very strong correlation, 0.70–0.89 a strong correlation, 0.50–0.69 moderate, 0.26–0.49 weak and 0.00–0.25 little if any correlation [12]. The Bland & Altman method was used to examine heteroscedasticity and potential systematic biases between the CAS and EOS measurements [5]. When zero lies within the 95 % CI, no bias exists between the measurements [34]. For the Bland & Altman method, the mean VV angles of the CAS and the EOS measurements were calculated. The mean differences between the CAS and EOS measurements were also calculated by subtracting the angle measured by the EOS system from the angle measured by CAS. Cohen’s *κ* coefficients were calculated to investigate agreement in the number of outliers as measured with CAS and EOS [11]. A deviation of >3° varus or valgus from the neutral axis was considered an outlier [20]. The *κ* values were interpreted according to Landis and Koch [24]: <0 represents less than chance agreement, 0.01–0.20 slight agreement, 0.21–0.40 fair agreement, 0.41–0.60 moderate agreement, 0.61–0.80 substantial agreement and 0.81–0.99 almost perfect agreement. χ^2^ tests were performed to assess statistically significant differences in the number of outliers. For all statistical analyses, a *P* value of <0.05 was considered to indicate statistical significance.

## Results

**Fig. 1:**
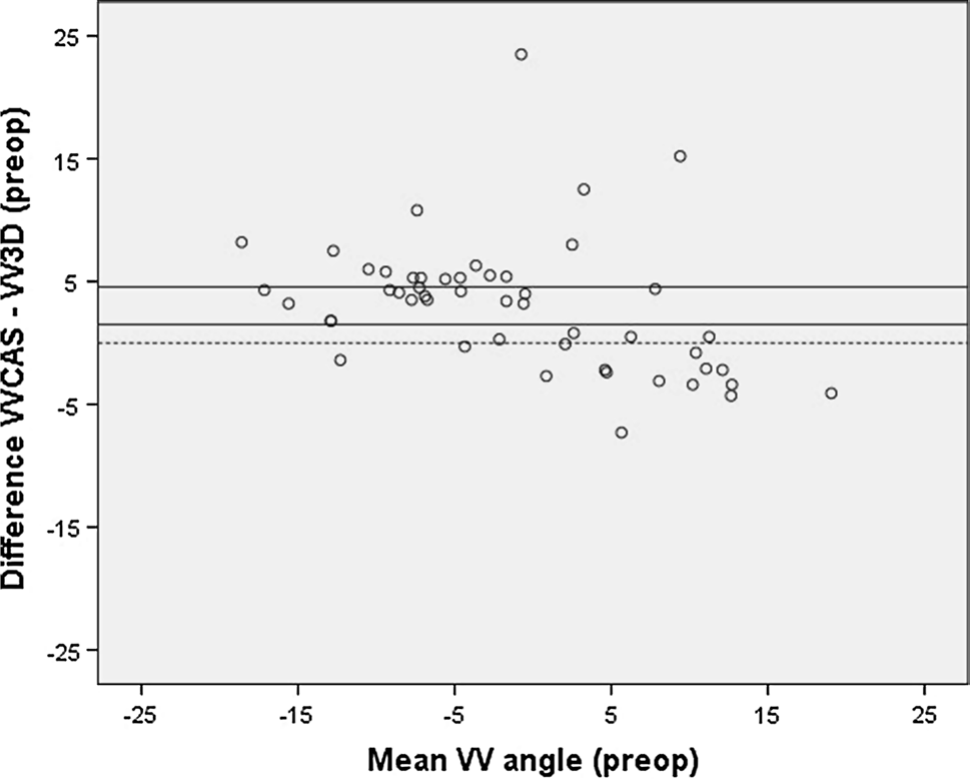
Bland & Altman *plot* of the primary CAS measurement and preoperative EOS measurement of the varus/valgus angle, showing heteroscedasticity

**Fig. 2:**
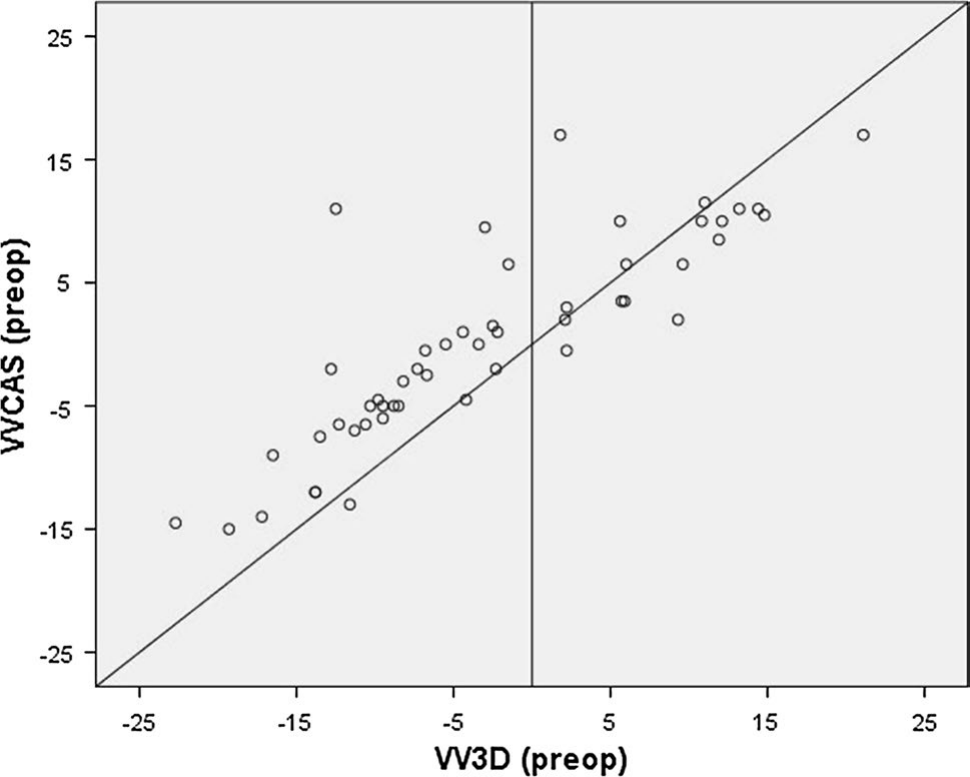
For varus legs, EOS measures more varus, and for valgus legs it measures more valgus than CAS

When the CAS measurements were compared with the preoperative EOS measurements, there was a significant difference between the VV angle measured using CAS (VVCAS) and measured using EOS (VV3D) (Table 1). The Bland & Altman plot showed heteroscedasticity (Fig. 1). This means that for varus legs the EOS measures a larger varus angle, and for valgus legs it measures a larger valgus angle than CAS (Fig. 2). Correlation between the two measurement techniques was strong, and the *κ* coefficient showed fair agreement on number of outliers (Table 1).

**Table 1:** Comparison of CAS and EOS measurements

	Mean	SD	Mean difference (95 % CI)	SDΔ	Range of difference CAS-EOS	*P*-value	Spearman’s *ρ*	*κ*
*Before implantation of prosthesis*								
VVCAS	0	8.3						
VV3D	−3	10.3	3 (1.5– 4.6)	5.4	−7 to 24	≤0.001*	0.87	0.34
mLDFA CAS	2	3.9						
mLDFA EOS	1	2.8	1 (−0.2–1.3)	2.6	−7 to 6	0.12	0.76	0.58
mMPTA CAS	−2	6.3						
mMPTA EOS	−4	5.5	2 (0.4–3.3)	4.8	−9 to 15	0.01*	0.67	0.44
*After implantation of prosthesis*								
VVCAS	0	3.7						
VV3D	−2	3.3	2 (1.2–3.3)	3.6	−2 to 21	≤0.001*	0.68	0.19

For calculating the mean difference, the angle measured by the EOS system was subtracted from the CAS angle

SD = standard deviation; 95 % CI = 95 % confidence interval; CAS = computer-assisted surgery; VVCAS = varus/valgus angle measured using CAS; VV3D = varus/valgus angle measured in 3D using EOS; mLDFA = mechanical lateral distal femoral angle; mMPTA = mechanical medial proximal tibial angle

* Statistical significance (*P* < 0.05)

**Fig. 3:**
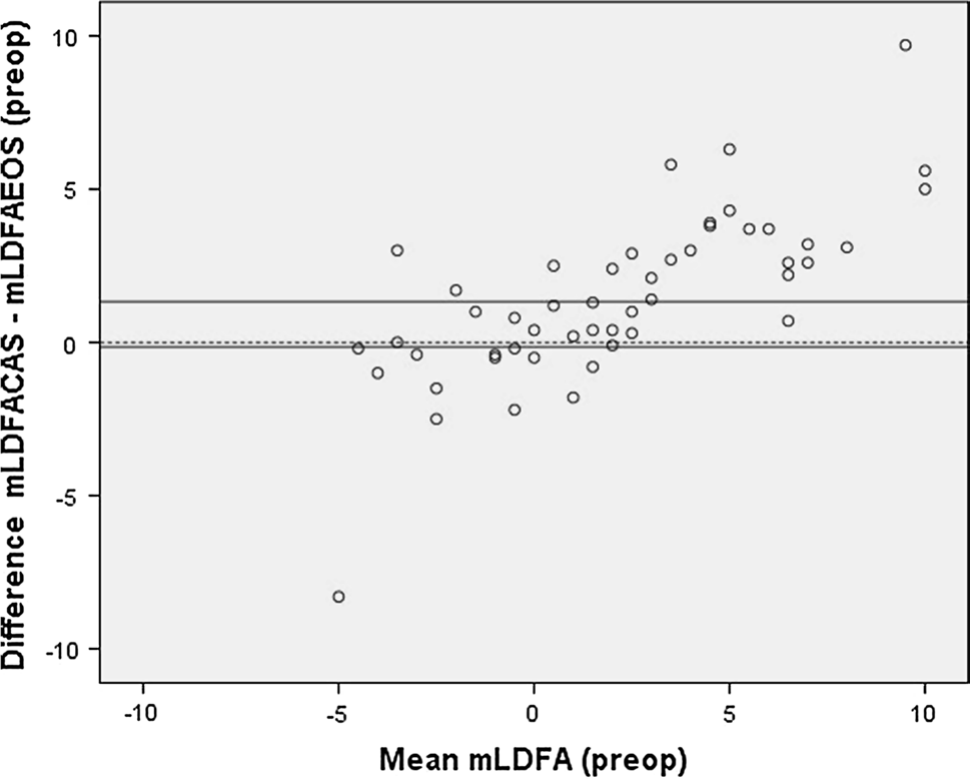
Bland & Altman *plot* of the primary CAS measurement and preoperative EOS measurement of the mechanical lateral distal femoral angle, showing no systematic bias

**Fig. 4:**
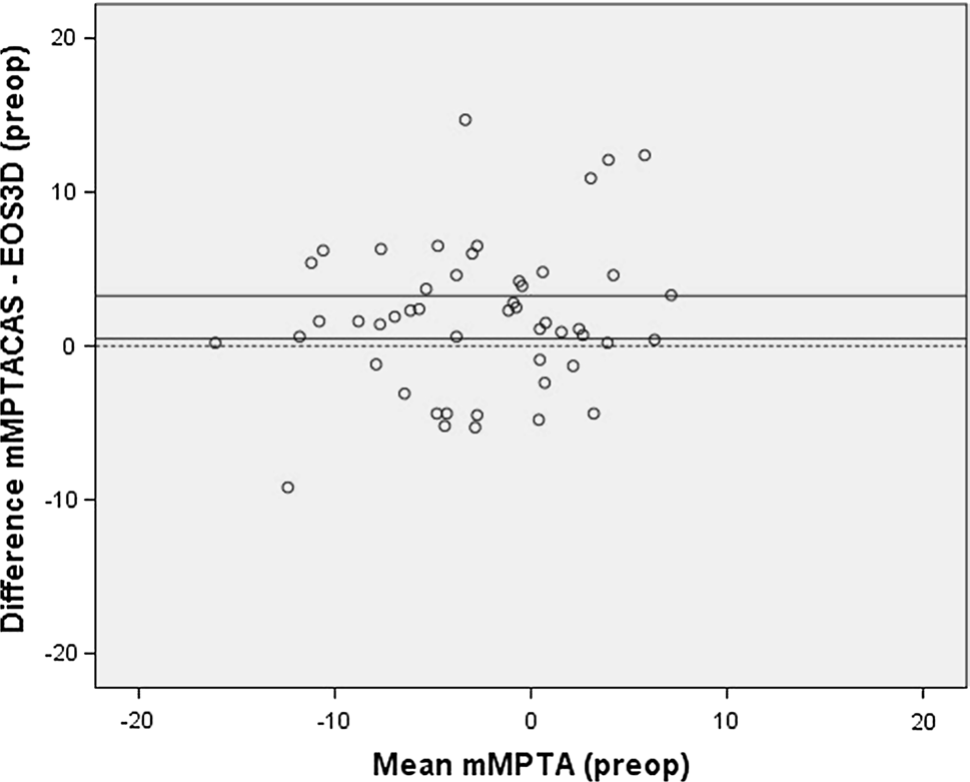
Bland & Altman *plot* of the primary CAS measurement and preoperative EOS measurement of the mechanical medial proximal tibial angle, showing a systematic bias

There was no significant difference and no systematic bias (Fig. 3) between the mLDFA measured using CAS and EOS (Table 1). Correlation between the CAS and EOS measurements was strong, and there was moderate agreement on the number of outliers (Table 1). A significant difference was found between the measurement of the mMPTA using CAS and EOS (Table 1). CAS was measuring more valgus; this was confirmed with a systematic bias using the Bland & Altman method (Fig. 4). Correlation between the two measurement techniques was moderate, and the *κ* coefficient showed a moderate agreement on number of outliers (Table 1).

**Fig. 5:**
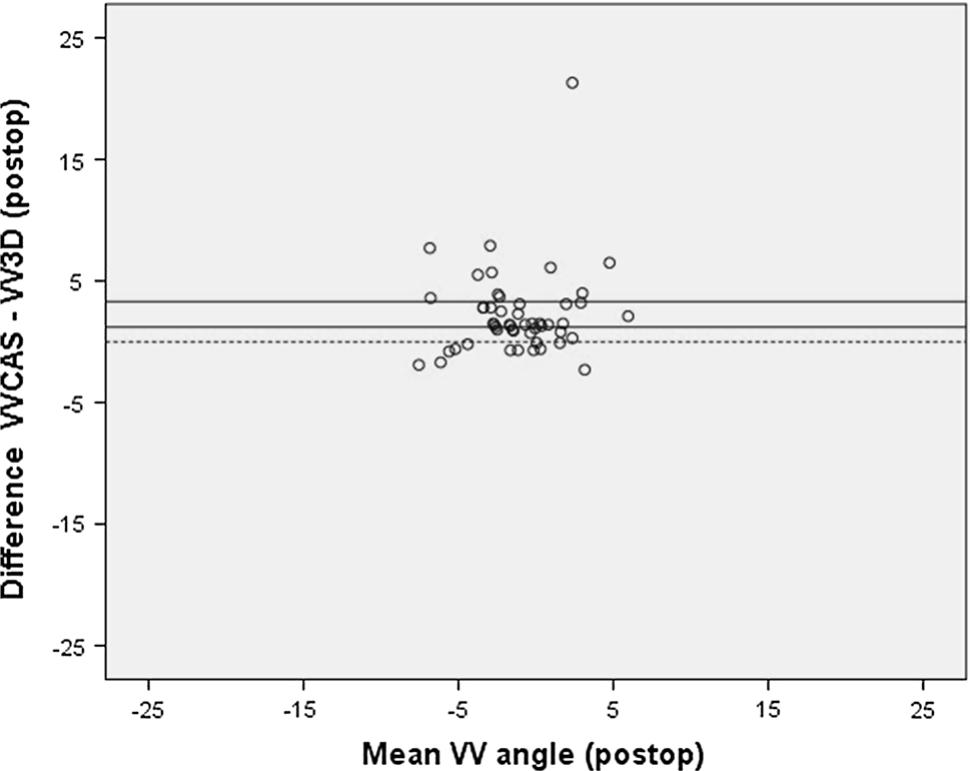
Bland & Altman *plot* of the second CAS measurement and postoperative EOS measurement of the varus/valgus angle, showing a systematic bias

When the second VVCAS measurement was compared to the postoperative VV3D measurement, a significant difference was found (Table 1). The Bland & Altman plot showed that the CAS systematically measured more valgus than the EOS (Fig. 5). Correlation between the CAS and EOS measurements was moderate, and the *κ* coefficient showed slight agreement on number of outliers (Table 1).

## Discussion

The most important finding of the present study was that the intraoperative CAS measurements during TKA differed from almost all EOS 3D pre- and postoperative coronal alignment measurements. VV measurements using CAS measured a smaller angle for both varus and valgus legs when compared to the preoperative EOS measurements. CAS showed a significantly larger valgus angle than the preoperative EOS 3D measurement of the mMPTA. The preoperative measurement of the mLDFA did not show any significant difference. VV measurements of CAS compared to the postoperative EOS measurements had significantly more valgus.

Previous studies have shown discrepancies between intraoperative CAS measurements and pre- and postoperative alignment measurements [1, 18, 37, 42, 43]. Several potentially explanatory factors have been mentioned for this difference: the influence of malpositioning during acquisition of LLRs on alignment measurements, the validity and reliability of alignment measurements on LLRs, the influence of a weight-bearing position on alignment measurements, and errors in the validity and reliability of CAS measurements. In previous studies comparing CAS measurements with radiographic measurements, malpositioning during acquisition and leg deformity have been one of the main explanations for the differences found. In this study, however, EOS 3D reconstructions were used to measure alignment thus eliminating potential bias caused by leg deformity or malpositioning [41]. This phenomenon is also shown in an experiment conducted by Meijer et al. [30], where an artificial leg containing a knee prosthesis was placed in several different positions. LLRs were made, and 2D measurements and 3D reconstructions were performed for these different positions. It was concluded that 2D alignment measurements differed considerably from the preset angle of the artificial leg, while the 3D reconstructions showed small deviation [30]. Besides validity, excellent intra- and interobserver reliability has been shown in the same study when performing knee prosthesis alignment measurements using EOS 3D reconstructions [29].

The difference between the supine and weight-bearing position of the patient may be an important reason for measurement differences. Coronal alignment of the knee is a dynamic parameter that can be influenced by both a weight-bearing position and the amount of flexion in the knee. Three studies [7, 36, 38] have compared alignment measurements in supine and weight-bearing position, finding significant differences between the two measurement methods. Brouwer et al. [7] and Specogna et al. [38] found an average of, respectively, 2° and 1.5° more varus in the weight-bearing position than in the supine position. Yet these studies only included knees with a varus deformity. Sabharwal et al. [36] found that patients with a substantial mechanical axis deviation were more likely to show differences in outcome of measurements in supine and weight-bearing position. This may also be the reason why the EOS measurements showed a larger varus angle for varus legs and a larger valgus angle for valgus legs compared to the supine CAS measurements. Overestimation of the VV angle on LLRs was also reported in three other studies comparing CAS and radiographic measurements [37, 42, 43]. This effect for the postoperative EOS measurements was not found in the present study. It is our hypothesis that after implantation of the prosthesis substantial mechanical axis deviations and ligamentous imbalances were corrected. The effect of a weight-bearing position is most distinct for larger VV angles and laxity of collaterals.

The validity and reliability of CAS measurements may play an important role in the measurement differences. Hauschild et al. [17] reported that alignment measurements using CAS are highly valid, but these measurements are prone to error when the knee is flexed. A cadaveric study investigating intraobserver errors when obtaining visually selected anatomical landmarks showed a maximum error of the VV of 1.3°, but this was done on bone stripped of all soft tissue, making it easier to register the landmarks [44]. A second study conducted by the same research group showed an error of 0.7° for the VV and also found low reliability of the registration of anatomical landmarks and significant interobserver differences [45]. A study comparing CAS, LLR and CT measurements found that LLR and CT correlated well, but CAS did not correlate well with LLR or CT. This raises the question about the reliability of intraoperative CAS measurements [1]. Intraoperative changes, such as movement of the trackers, may also be of influence on the CAS measurements. Although these studies report on the results of imageless CAS systems, none investigated the specific CAS system used in the present study. Reliability and validity may also be dependent on the design and software of a specific system; hence, it can be questioned whether results of studies on other systems are applicable to the system used in the present study.

It is suggested that correlation between CAS and radiographic measurements after TKA may be influenced by the moment of acquisition of the postoperative radiographs. Hauschild et al. [18] compared two groups that underwent CAS-TKA. One group received LLRs 2 weeks postoperatively and the other group 3 months postoperatively. Correlations between radiographic measurements using CAS and LLRs taken 3 months postoperatively were excellent, but were poor when the intraoperative CAS measurements were compared with alignment measurements performed on LLRs taken 2 weeks postoperatively. They hypothesised that after 3 months patients are usually able to bear full weight and full or near full extension of the knee, which improves correlation between alignment measurements using CAS and postoperative LLRs. The moment of assessment of the postoperative LLRs may thus be of influence. However, the fact that an LLR is made when applying full weight-bearing would theoretically cause a larger difference between CAS and LLR measurements instead of a smaller one, as CAS measurements are non-weight-bearing. Also, the conclusions of the study of Hauschild et al. [18] were drawn from a comparison between two patient samples so the differences found between the two acquisition moments may not be based on time but on patient factors. In this study, postoperative LLRs were taken 6 weeks postoperatively, at which point patients are generally able to apply full weight on their operated leg and can extend the knee. Moreover, the EOS system corrects malpositioning during acquisition, including flexion of the knee [30]; therefore, the moment of acquisition is not expected to influence our results.

This study has some limitations. First of all, the LLR measurements were performed by a single observer. However it should be noted that this observer has extensive experience in performing EOS 3D reconstructions. Moreover interobserver reliability of EOS 3D measurements has proven to be excellent [29]. Secondly, a potential bias might be present during the CAS measurements. When performing preoperative planning, leg alignment measurements are taken and the first intraoperative CAS measurements cannot be blinded, as that is not possible in this setup. The orthopaedic surgeon might therefore be potentially biased when performing the first CAS measurement. Although the second CAS measurement was not blinded either, measurement bias is unlikely as the outcome of postoperative EOS measurements during TKA is not known. Thirdly, EOS imaging is a standing procedure without true information about the amount of weight-bearing on each leg. The expectation is that patients are grossly dividing their weight equally between both legs, but this is not known for sure. Therefore, when comparing EOS with CAS and attributing differences to the fact that CAS is not weight-bearing, it is not possible to know exactly the amount of forces influencing these measurements.

Eventually the results of this study are of clinical relevance, since it raises concerns regarding the validity and reliability of CAS systems in TKA.

## Conclusion

The results of this study indicate that differences in alignment measurements between CAS and pre- and postoperative LLRs are mainly due to the variance between weight-bearing and non-weight-bearing positions, and might also be caused by potential errors in validity and reliability of the CAS system. Surgeons should be aware of these measurement differences and the pitfalls of both measurement techniques. It is not advised to rely solely on CAS measurements during CAS-TKA.

## Figures and Tables

**Fig. 6:**
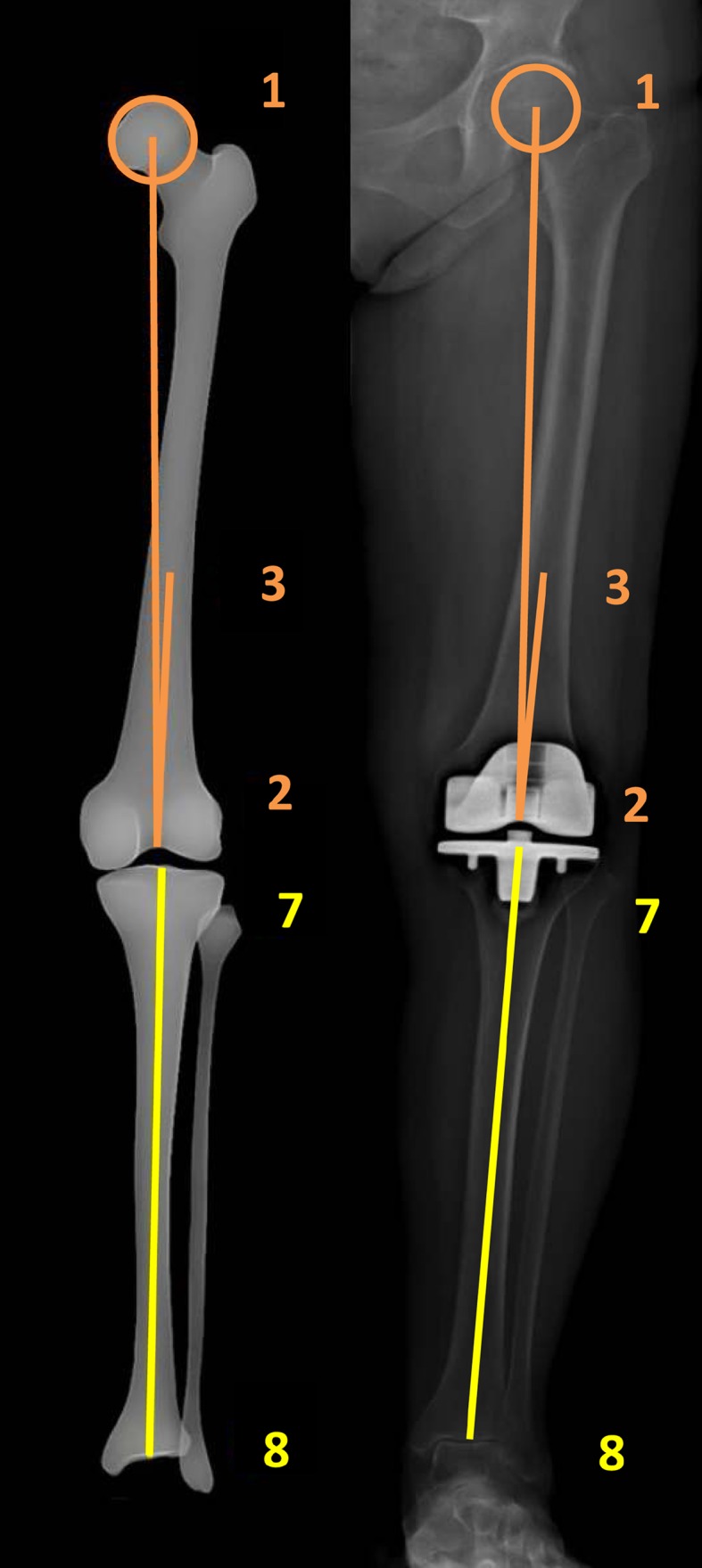
(*left*) Identification of the landmarks on the AP images

**Fig. 7:**
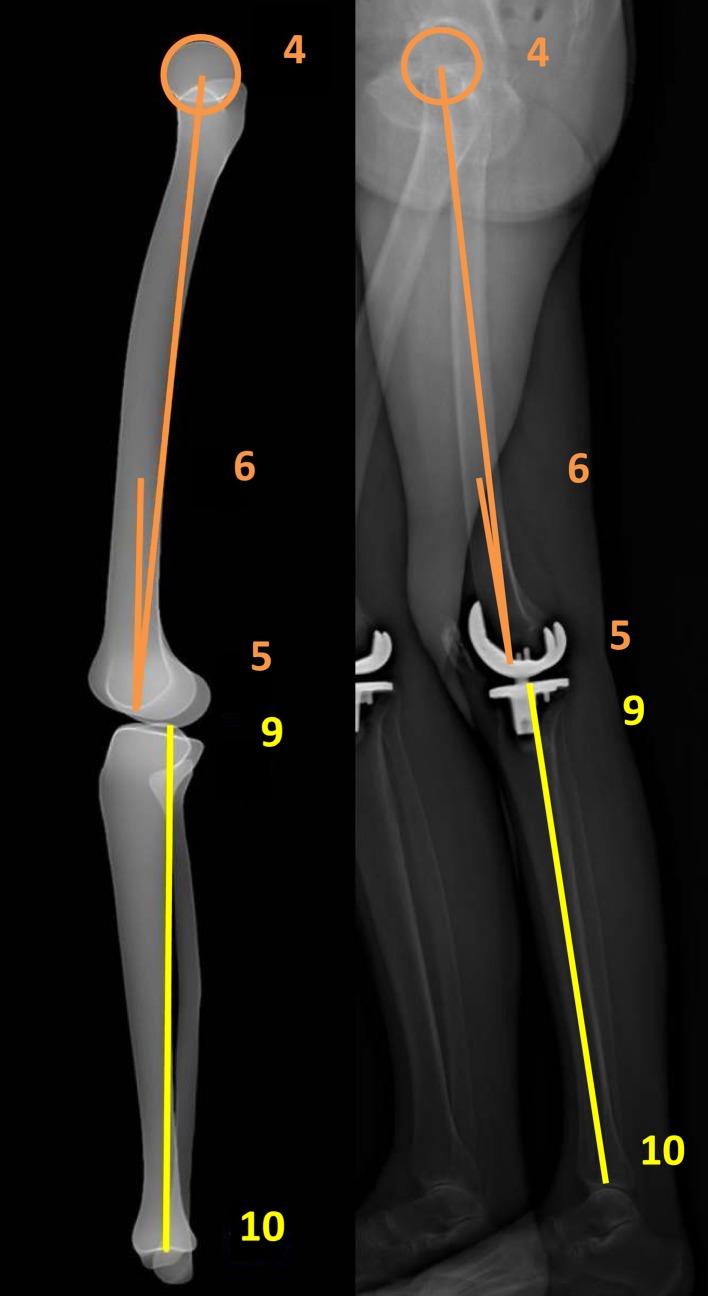
(*right*) Identification of the landmarks on the LAT images

**Fig. 8:**
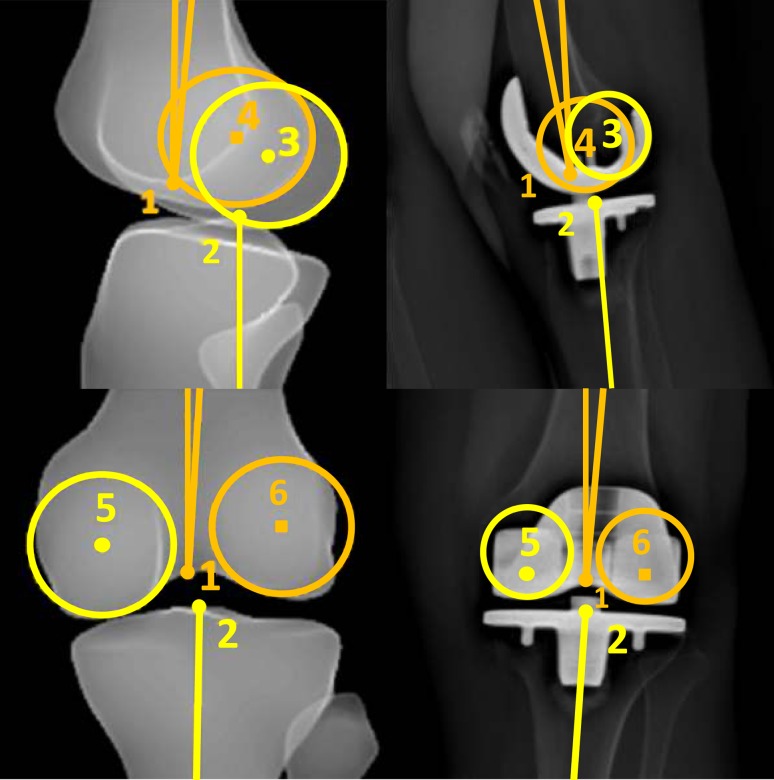
Adjustment of the landmarks on the AP and LAT images

## Appendix

For 3D measurements on lower limbs without a knee prosthesis, the “full 3D” mode was chosen. First, identification of the lower limb was performed in ten steps (Figs. 6, 7):

### Identification of femur

- Centre of femoral head (point 1 and 4),
- Centre of notch (point 2 and 5),
- Centre of diaphysis in its upper third (point 3 and 6).

### Identification of tibia

- Centre of tibial spines (point 7 and 9),
- Centre of distal articular surface (point 8 and 10).

The next step is adjustment of the landmarks in four steps (Fig. 8):

1. Adjustment of the position of the sphere of the femoral head in both views. It is possible to enlarge or minimise the size of the sphere according to the size and shape of the femoral head, in order to mark the centre of the femoral head as precisely as possible;
2. Adjustment of the point in the centre of the distal third of the diaphysis of the femur;
3. Adjustment of the position of the point in the centre of the femoral notch and tibial plateau, and marking of the femoral condyles. The condyles have to be identified on the AP and LAT images using the two spheres. It is possible to adjust the size of the spheres, according to the size of the condyles. On the AP image, the centre of the spheres has to be located in the centre of each condyle. On the LAT image, the spheres have to be tangent to the posterior part of the condyles. It is important not to confuse the medial with the lateral condyles. In order to identify the right condyle, the epipolar line is used to differentiate between the two condyles by observing the correspondence of condylar height on both the AP and the LAT image;
4. Adjustment of the reference point in the centre of the distal articular surface on the AP and LAT images.

Then, several anatomical landmarks of the femur and tibia are marked:

- The centre of the upper and lower section of the femoral neck,
- The proximal and distal medial and lateral edge of the diaphysis of the femur,
- The posterior edge of the internal and external tibial plate.

Based on the anatomical landmarks, an envelope of the femur and tibia is developed. This envelope can be adjusted to the bony landmarks if necessary. After accepting the suggested envelope, the software calculates the alignment angles.

Since several landmarks disappear or change when a knee prosthesis is in situ, the “lower limb alignment” mode was chosen. The observers made the following agreements on marking the landmarks:

- Instead of the centre of tibial spines, the centre of the tibial plateau is chosen;
- Instead of marking the distal femoral notch, the centre of the femoral component is marked;
- Instead of marking the anatomical femoral condyles, the condyles of the femoral component are marked.

In order to calculate coronal and sagittal alignment parameters of the lower limb in 3D, the “lower limb alignment” mode is used. The first step is to define the left or right lower limb and to choose the modelling “lower limb alignment” mode. Next, identification of the lower limb on the AP and LAT images is done in ten steps (Figs. 6, 7):

### Femur

- Centre of femoral head (points 1 and 4);
- Centre of the distal femoral notch (points 2 and 5);
- Centre of the diaphysis in its distal third (points 3 and 6).

### Tibia

- Centre of the tibial spines. When a knee prosthesis is in situ, the tibial spines disappear; therefore, the centre of the tibial plateau is chosen, and the axis from the centre of the ankle to the centre of the tibial plateau represents the anatomical axis of the tibia (points 7 and 9);
- Centre of the distal articular surface in the upper ankle joint (points 8 and 10).

The next step is adjustment of the landmarks in four steps (Fig. 8):

5. Adjustment of the position of the sphere of the femoral head in both views. It is possible to enlarge or minimise the size of the sphere according to the size and shape of the femoral head, in order to mark the centre of the femoral head as precisely as possible;
6. Adjustment of the point in the centre of the distal third of the diaphysis of the femur;
7. Adjustment of the position of the point in the centre of the femoral notch and tibial plateau, and marking of the femoral condyles. The condyles have to be identified on the AP and LAT images using the two spheres. It is possible to adjust the size of the spheres, according to the size of the condyles. On the AP image, the centre of the spheres has to be located in the centre of each condyle. On the LAT image, the spheres have to be tangent to the posterior part of the condyles. It is important not to confuse the medial with the lateral condyles. In order to identify the right condyle, the epipolar line is used to differentiate between the two condyles by observing the correspondence of condylar height on both the AP and the LAT image;
8. Adjustment of the reference point in the centre of the distal articular surface on the AP and LAT images.

VV2D is the angle between the mechanical axis of the femur (axis between points 1 and 2) and the tibia (axis between points 7 and 8) on the AP image (Fig. 6). For the 3D measurement, the points marked on the AP (Fig. 6) and LAT (Fig. 7) images as described above are combined to generate the mechanical axes of femur and tibia. VV3D is the angle between the three-dimensional mechanical axis of the femur (axis between points 1–4 and 2–5) and tibia (axis between point 7–9 and 8–10). A positive value indicates valgus, and a negative value indicates varus.
